# Knockout Mice: Is It Just Genetics? Effect of Enriched Housing on Fibulin-4^+/−^ Mice

**DOI:** 10.1371/journal.pone.0000229

**Published:** 2007-02-21

**Authors:** Elizabeth Cudilo, Hamda Al Naemi, Lihua Marmorstein, Ann L. Baldwin

**Affiliations:** 1 Department of Physiology, College of Medicine, University of Arizona, Tucson, Arizona, United States of America; 2 Biological Sciences, University of Qatar, Doha, Qatar; 3 Department of Ophthalmology and Vision Science, University of Arizona, Tucson, Arizona, United States of America; Baylor College of Medicine, United States of America

## Abstract

**Background:**

Fibulin-4 is an extracellular matrix protein expressed by vascular smooth muscle cells that is essential for maintaining arterial integrity. Fibulin-4^−/−^ mice die just before birth due to arterial hemorrhage, but fibulin-4^+/−^ mice appear to be outwardly normal. Experiments were therefore performed to determine whether fibulin-4^+/−^ mice display arterial pathologies on a microscopic scale. After preliminary experiments were performed, a second purpose developed, which was to test the hypothesis that any observed pathologies would be ameliorated by housing the animals in enriched cages.

**Methodology:**

Fibulin-4^+/−^ and wild-type mice were housed either four/cage in standard cages or two per cage in larger cages, each cage containing a tunnel and a wheel. After three weeks the mice were sacrificed, and the aortas perfusion-fixed and excised for light and electron microscopy.

**Principle Findings:**

When the mice were in standard cages, localized regions of disorganized extracellular matrix and collagen fibers consistently appeared between some of the medial smooth muscle cells in the fibulin-4^+/−^ mice. In the wild-type mice, the smooth muscle cells were closely connected to each other and the media was more compact. The number of disorganized regions per square mm was significantly greater for fibulin-4^+/−^ mice (172±43 (SEM)) than for wild-type mice (15±8) (p<0.01, n = 8). When the mice were in enriched cages, the fibulin-4^+/−^ mice showed significantly fewer disorganized regions than those in standard cages (35±12) (p<0.05, n = 8). The wild type mice also showed fewer disorganized regions (3±2), but this difference was not significant.

**Conclusions:**

These results indicate that arterial pathologies manifested in fibulin-4^+/−^ mice can be reduced by enriching the housing conditions, and imply that appropriate environments may counteract the effects of some genetic deficiencies.

## Introduction

The mechanical properties of the vascular wall are mainly determined by elastin and collagen with a contribution from smooth muscle cells. Stress is uniformly distributed between these structures. Tension is transmitted between adjacent smooth muscle cells, and between smooth muscle cells and surrounding extracellular matrix (ECM) fibers by means of strong, compliant attachments mediated by a wide variety of proteins [1]. Approximately twenty years ago, a 6-member family of proteins was discovered: the fibulin family. These fibulins were hypothesized to function as intramolecular bridges, stabilizing the organization of other ECM structures, such as elastic and collagen fibers [2].

The various members of the fibulin family have somewhat different functions and locations. Fibulin-1 co-localizes with elastin and is found in the ECM surrounding arterial smooth muscle cells [3]. Fibulin-2 has been observed in coronary arteries and the aortic arch, binding to elastin and fibrillin-1 [4], but fibulins-3 and 6 have not been seen in large blood vessels. Fibulin-4 is localized in the aortic media and is essential for maintaining arterial integrity since homozygous fibulin-4 knockout mice die just before birth due to arterial hemorrhage. In these mice, elastin cross-links are diminished and the elastic lamellae are fragmented [5]. Fibulin-5 is produced at low levels by vascular smooth muscle cells and is localized to the surface of elastic fibers [6]. Homozygous mutation in fibulin-5 is associated with a form of recessive *cutis laxa*, recognized in humans by sagging skin and a tortuous aorta. Recently, fibulin-4 (FBLN4) has also been linked to an autosomal recessive form of *cutis laxa* [7]. In this case, the patient exhibited vascular tortuosity and ascending aortic aneurism.

Although it is clear that fibulin-4 is essential for maintaining vascular integrity, it is not known whether heterozygous fibulin-4 mice, which appear outwardly healthy and have normal heart rate and blood pressure, show arterial defects. Other studies have shown that mice only carrying one copy of a gene can exhibit deleterious effects. For example, mice deficient in both copies of a neuro-developmental gene, called neuroD2, die within a few weeks of birth, because the amygdala does not develop properly. However, mice carrying one copy of the gene show an impaired ability to form emotional memories and conditioned fear [8].

The first purpose of this study was to determine whether mice carrying only one copy of the fibulin-4 gene show aortic defects. It is thought that the development of mouse models deficient in one or more fibulins will facilitate the understanding of the biological functions of fibulins and the pathogenesis of associated diseases [9]. During preliminary experiments a second purpose also developed, which was to test the hypothesis that any observed pathological differences would be ameliorated by enriched housing conditions.

## Materials and Methods

### Procedure

Thirteen male wild type and 17 fibulin-4^+/−^ mice (3 months old) were used. As described previously [5], germ line-transmitting dimeric mice generated from embryonic stem cells containing the lacZ-neo cassette in exon-4 of the mouse fibulin-4 locus, were bred with C57BL/6 mice to produce the fibulin-4^+/−^ mice. The mice were genotyped by extracting DNA from tail biopsy samples [5]. Sixteen of the mice (7 wild type and 9 fibulin-4^+/−^) were housed in four standard cages (4 animals per cage) for one month prior to surgery. The remaining 14 (6 wild type and 8 fibulin-4^+/−^) were housed, at the same time, in four larger, enriched cages (2 animals per cage). The following protocols were reviewed and approved by the Institutional Animal Care and Use Committee, University of Arizona.

#### Cages

The standard cages were 26 cm (length)×16 cm (width)×12 cm (height) and contained only bedding. The food tray projected into the cage, leaving only a space of 4 cm between the tray and the floor at the lowest point of the tray. These cages are used routinely in the facility to house mice. The enriched cages were larger (33 cm (length), 25 cm (width)×25 cm (height), and contained a shelf, ladder and an exercise wheel. In addition, a plastic tube was placed inside each cage.

#### Monitoring Behavior

The mice housed in enriched cages were observed at night, some time between 20:30 and 22:00, on four occasions during the 3-week period. Each mouse was videotaped, using an infrared camera, for 2 minutes on each occasion and the tapes were later analyzed to determine the percentage of time that the mice used the exercise wheel.

#### Surgery

Each mouse was weighed and anesthetized with an anesthetic consisting of 0.9 ml 80 mg/ml ketamine and 0.1 ml of 10 mg/ml xylazine. (0.2 ml/100 g body weight). This anesthesia took about 20 minutes to take effect and kept the mouse at sufficient depth of anesthesia for another 20 minutes. A booster dose, prepared from 0.1 ml of 80 mg/ml ketamine and 1ml of phosphate-buffered saline, pH 7.4 (PBS) was given in 0.1 ml aliquots every subsequent 20 minutes. Usually only one booster dose was required. The mouse was positioned, supine, on the surgical tray with the paws taped to the tray to prevent rolling. Next, the abdomen was slit along the *linea alba* and the abdominal aorta was exposed. Angled Dietrich bulldog clamps were used to keep the abdomen open. The small intestine was retracted and covered with moist gauze, so as to expose the abdominal aorta and inferior vena cava. Then the vena cava was carefully separated from the abdominal aorta by blunt dissection, under stereo-microscopy. After the vessels were stripped of adipose tissue, two loose ties were placed around both vessels distal to the diaphragm. The most distal thread was double-tied securely around both vessels and a pair of angled Dietrich bulldog clamps was attached to the thread to provide a small amount of tension. A B-3 micro clamp was positioned, using forceps, at the proximal end of the exposed aorta, just below the diaphragm. Using the curved shanks to raise the aorta by its fat sheath, a small, 60° V-shaped incision was made between the two distal ties using mini-Vannas-style spring scissors. If the proximal aorta was clamped correctly, only a small drop of blood was lost from the aorta. A PBS-filled cannula was inserted through the incision, using curved shanks to lift up the tiny lip on the aorta created by the V-shaped incision, and slid gently up the vessel just past the loose distal tie. This tie was then tightened to secure the cannula and the B-3 micro clamp was removed. A small amount of back flow of blood down the cannula, indicated a successful cannulation. Next, an incision was made in the vena cava at approximately the level at which the aortic clamp had been placed, to act as an outlet. After the vena cava was cut, PBS was gently perfused, at 40 mm Hg pressure, through the aortic cannula, using a syringe. After perfusion of about 10 ml PBS, the fluid from the vena cava ran clear and the animal expired. At this point, the PBS-filled syringe was replaced with a syringe containing Karnovsky's fixative, pH 7.4. The aorta was flushed with 3 ml of fixative before replacing the B-3 micro clamp at the proximal end of the aorta and maintaining a fixation pressure of 40 mmHg. A piece of gauze soaked in fixative was applied over the vessel. After one hour of fixation the aorta was excised, cut transversely into two segments and fixed for a further hour in Karnovsky's fixative. In the case of aortas from 5 wild-type and 5 fibulin-4^+/−^mice, one segment was processed for light microscopy and the other for electron microscopy. All other aortas were just processed for electron microscopy

#### Preparation of Tissue for Light Microscopy

Aortic segments, fixed with Karnovsky's fixative, were embedded in wax [10]. The embedded tissue samples were thick sectioned (6 um sections) and then stained with Picro-Ponceau and Hematoxylin in order to identify collagen and elastin. The Picro-Ponceau was prepared by mixing 10 ml of 1% aqueous Ponceau S, 86 ml of saturated aqueous picric acid, and 1% aqueous acetic acid. The sections were examined and the arterial wall thickness measured by an investigator who was blinded to the experimental conditions.

#### Preparation of Tissue for Electron Microscopy

The aortic segments were rinsed in 0.2 M sodium cacodylate buffer, postfixed in osmium tetroxide, dehydrated with increasing concentrations of ethanol, and embedded in Spurrs resin for electron microscopy. The segments were oriented in the embedding medium so that they would be sectioned perpendicular to their longitudinal axis. The sections were collected on grids (3 grids for each aorta), that were identified with grid numbers, and stained with uranyl acetate and lead citrate which are general purpose stains for membranous structures and nucleic acids. The grids were examined under a Phillips CM12 Transverse Electron Microscope at a magnification of ×3000. For each grid, 50 fields of view were systematically recorded by scanning the sections from left to right and top to bottom. The investigator examining the grids was blinded to the experimental conditions.

### Statistical Analysis

Animal weights were compared using the Student t-test after establishing that the data passed the tests of normality and equal variance. The same procedures were performed for medial and lamella thicknesses. Numbers of gaps were compared using the Mann-Whitney Rank Sum test because not all data groups passed the normality test.

## Results

### Animal Weight

Both the wild-type and fibulin-4^+/−^ age-matched mice were significantly lighter when housed in the enriched cages compared to the standard cages (p<0.001). In enriched cages, the average weights of wild-type and fibulin-4^+/−^ mice were 25.7 g±4.7 (SD) and 29.4 g±4.8, respectively (no significant difference). Corresponding weights when the mice were housed in standard cages were 43.9 g±4.9 (SD) and 42.9 g±7.5. In addition, mice housed in the standard cages showed very large quantities of adipose fat around the aorta, whereas those housed in the enriched cages did not. This result was probably due to the presence of the exercise wheel in each enriched cage (see below, *‘Animal Behavior’*). When the mice were observed at night, the wheel was in use for a large percentage of the time, whereas the mice in the standard cages mainly assumed a fairly stationary upright position in the corners of the cages.

### Animal Behavior

On average, the mice spent 40% of the observation time exercising in the wheel. The percentages ranged from 22% to 62% and there was no significant difference in behavior between the wild-type mice and the fibulin-4^+/−^ mice.

### Light Microscopy

**Figure 1:**
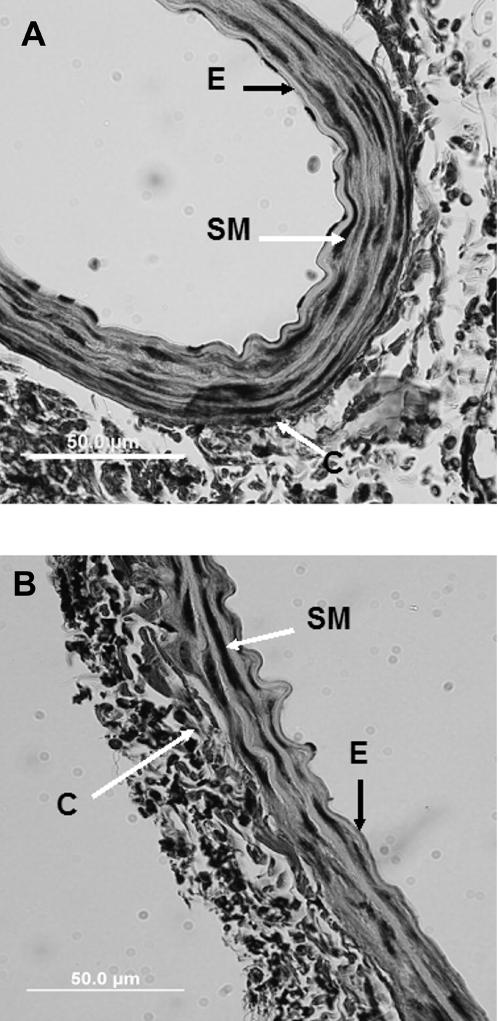
Light micrographs of transverse section of mouse aorta from wild-type (panel A) and fibulin-4^+/−^ (panel B). Sections were stained for collagen (C), elastin (E) and smooth muscle cell nuclei (SM).

Under light microscopy (×400 magnification), the aortic sections appeared to be structurally very similar to each other, whether the mice were housed in standard or enriched cages (figure 1). Four or five elastic lamellae were visible in the media, separated by single layers of smooth muscle cells. Collage fibers were present mainly in the adventitia. The mean medial thickness (derived from 4 measurements on 3 sections from each vessel) of the aortic media from wild-type rats housed in standard cages was 26.34 µm±7.37 (SD) compared to 21.95 µm±5.83 (SD) for similarly housed Fibulin-4^+/−^ mice. Corresponding values for the mean thickness of individual lamellae were 2.47 µm±0.40 (SD) and 2.44 µm±0.48. There was no significant difference between the wild-type mice and the fibulin-4^+/−^ mice in either case.

### Electron Microscopy

**Figure 2:**
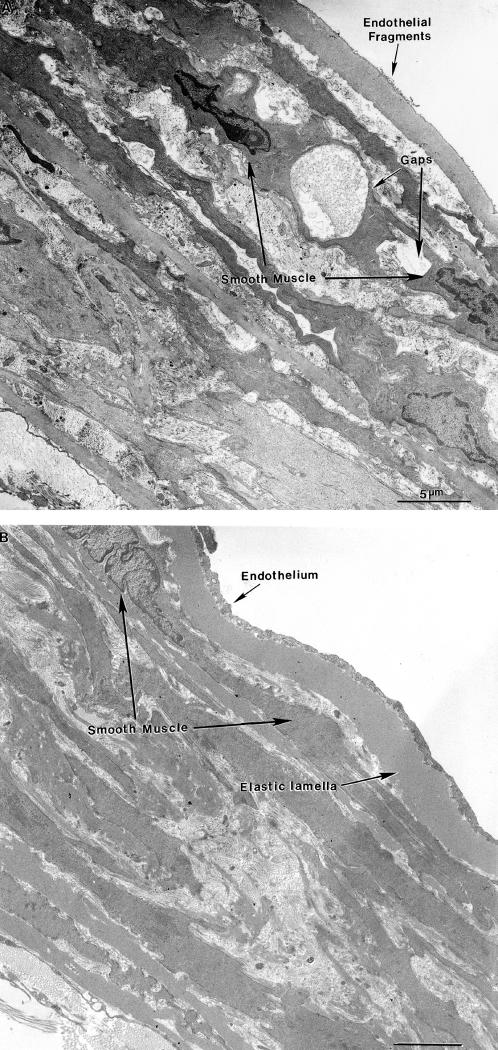
Electron micrographs of transverse ultra-thin sections of aortas from fibulin-4^+/−^ mice housed in a standard cage (panel A) and an enriched cage (panel B).

**Figure 3:**
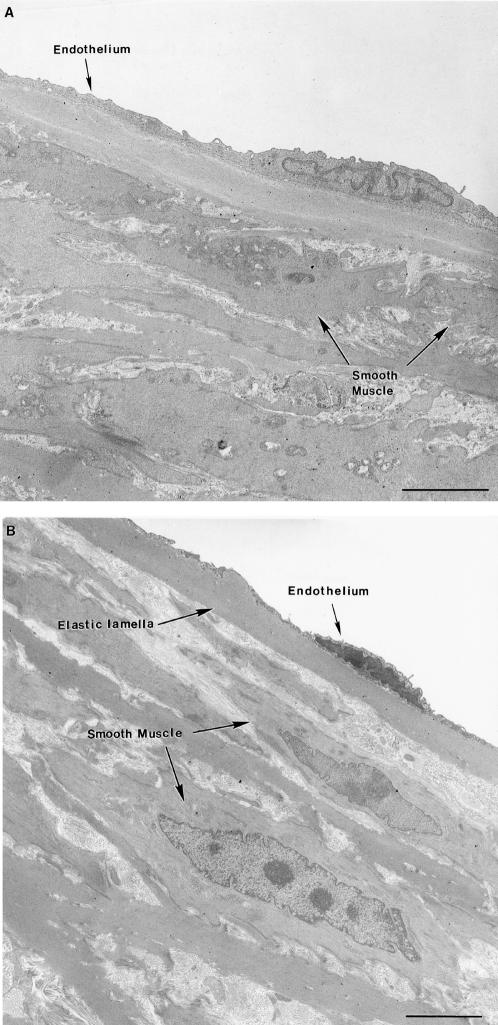
Electron micrographs of transverse ultra-thin sections of aortas from wild-type mice housed in a standard cage (panel A) and an enriched cage (panel B).

When the structural appearance of the aortas was compared under transmission electron microscopy (magnification×3,000) one consistent difference was observed between aortas from the wild-type (n = 7) and the fibulin-4^+/−^ mice (n = 9) housed in standard cages. Localized regions of disorganized extracellular matrix and collagen fibers appeared between 10%–15% of the medial smooth muscle cell junctions in the fibulin-4^+/−^ mice (figure 2A). These regions are referred to as ‘gaps’. The gaps were identified by their position (within smooth muscle cell-cell junctions), circular shape, size (3–6 µm in diameter), and the fact that they were bordered by cytoplasmic projections that extended from the otherwise separated adjacent smooth muscle cells to form the only intact parts of the tight junction. These findings are consistent with the proposed function of fibulin-4, to stabilize the organization of ECM structures. In the wild-type mice the smooth muscle cells that were closely apposed were almost all fully connected by tight junctions. In addition, the aortic media was more compact (figure 3A). The number of gaps per square mm was significantly greater for fibulin-4^+/−^ mice (by a hundred-fold) than for wild-type mice (p = 0.006) (Table 1). These parameters were obtained by averaging counts from 50 fields of view of 3 grids supporting sections from each vessel. Another difference between the two strains was that the endothelium was often sloughed in the fibulin-4^+/−^ group, with just a few fibrous remnants adhering to the internal elastic lamella (figure 2A) whereas it was intact in the wild-type (figure 3A). Sometimes, in the fibulin-4^+/−^ mice, the endothelium was still present but was only attached to the internal elastic lamella at membrane associated dense plaques, with short pegs extending downwards to the elastic lamella. Such attachment structures have been described previously [11].

**Table 1:** Standard Housing (four per cage), Number of Gaps per square mm (Wild Type Mice versus Fibulin-4^+/−^ Mice)

Group	Wild Type	Fibulin-4+/−
Mean	15.00	172.00
SD	21.00	129.00
SEM	7.60	43.00
N	7	9

Statistical significance, p = 0.006 (Statistical significance obtained using Mann-Whitney Rank Sum Test)

In fibulin-4^+/−^ mice that were housed two per cage in the enriched cages, there was a significant decrease in the average number of gaps compared to that observed when they were housed in standard cages (p = 0.016) (Table 2). In addition, the endothelium was intact, similar to that observed in aortas from wild-type mice (figure 2B). There was no significant decrease in the average number of gaps in the wild-type mice when they were housed two per cage in the enriched cages, but there was a trend towards a lower number (Table 3). The structure of the aortas did not appear to be any different whatever the type of housing (compare figures 3A and 3B). When the average number of gaps was compared between the wild-type mice and the fibulin-4^−/+^ mice, both housed in enriched cages (Table 4), the wild-type still had significantly fewer gaps than the fibulin-4^+/−^ mice (p = 0.008). This was due to the statistically non-significant decrease in gap number when the wild-type mice were housed in the enriched cages.

**Table 2:** Fibulin-4^+/−^ Mice, Number of Gaps per square mm (Standard versus Enriched Housing)

Group	Standard	Enriched
Mean	172.00	35.00
SD	129.00	34.00
SEM	43.00	12.00
N	7	6

Statistical significance, p = 0.016 (Statistical significance obtained using Mann-Whitney Rank Sum Test)

**Table 3:** Wild Type Mice, Number of Gaps per square mm (Standard versus Enriched Housing)

Group	Standard	Enriched
Mean	15.00	3.00
SD	21.00	5.00
SEM	7.60	2.00
N	7	6

No significant difference (Statistical significance obtained using Mann-Whitney Rank Sum Test)

**Table 4:** Enriched Housing (two per cage), Number of Gaps per square mm (Wild Type Mice versus Fibulin-4^+/−^ Mice)

Group	Wild Type	Fibulin-4+/−
Mean	3.00	35.00
SD	5.00	34.00
SEM	2.00	12.00
N	6	8

Statistical significance, p = 0.008 (Statistical significance obtained using Mann-Whitney Rank Sum Test)

## Discussion

The evidence presented suggests that although fibulin-4^+/−^ mice are genetically predisposed to gap formation between aortic smooth muscle cells and to endothelial damage, simply housing the mice in an enriched environment can significantly reduce the gaps and maintain endothelial integrity. It is not clear why fibulin-4^+/−^ mice showed aortic endothelial damage when housed in standard cages. A previous study [12] showed that fibrillin-1 and fibulin-2 were co-localized in the same tissues of the body, and it was suggested that fibrillin-1 assumes the functions of fibulin-2 if the physiological conditions permit. It is possible that other members of the fibulin family may have fibulin surrogates. If so, perhaps housing the fibulin-4^+/−^ mice in the enriched environment creates the ideal conditions under which a surrogate protein for fibulin-4 can take over and ultimately inhibit gap formation.

This finding has two important implications. First, many investigators might not be aware that housing conditions can affect the differences that are observed between knock-out and wild-type strains. Thus, findings that are assumed to be due simply to genetic differences might be wrongly interpreted, and environmental factors may play an important role. Secondly, with regard to fibulin-4^+/−^ mice and to the noticeable appearance of gaps between smooth muscle cells only when these mice were housed in standard cages, it is possible that genetically induced weaknesses in arteries might be exacerbated under certain environmental situations. For example, in the standard cages the mice were not offered the opportunity to exercise or to climb, nocturnal behaviors that they repeatedly chose if the cages were suitably equipped with a wheel, shelf, ladder and tube.

Previous studies in the literature are consistent with our conclusion that observed differences between wild-type and knock-out mice are not solely due to genetic deficiencies, but may require a certain set on environmental conditions to make themselves manifest. Other authors have proposed that neither genetic nor environmental agents acting independently cause disease [13]. They encourage geneticists to consider disease as a consequence of interactions between factors encoded in the genotype and exposure to various environmental agents and situations. Evidence for this viewpoint is provided by some recent studies. Firstly, Crabbe et al [14] demonstrated that experiments characterizing the behavior of the same strains of mutant mice performed in three different laboratories (two in the USA and one in Canada) yielded results that were idiosynchratic to a particular laboratory. For example in one laboratory no difference was observed between 5-HT_1B_ null mutants and wild-type mice in distance traveled in an activity monitor, whereas there was a significant difference at the other two sites. Secondly, Lazarov et al [15] showed that exposure of transgenic mice to an enriched environment results in pronounced reductions in deposits of cerebral beta-amyloid peptides, a hallmark of Alzheimer's disease, compared to animals raised under standard housing conditions. Thirdly, a transgenic mouse model for Huntington's disease, a neurodegenerative disorder, showed delays in the onset of motor symptoms of the disease when the mice were housed with environmental enrichment [16]. The mice housed under standard conditions demonstrated severe reductions in brain-derived neurotropic factor in the hippocampus and striatum, which were entirely rescued by enrichment. In this case the enrichment consisted of small boxes, cylindrical tunnels and folded sheets of paper. These types of enrichment do not necessarily encourage increased exercise, in contrast to the exercise wheels provided in the present study. Thus exercise, per se, is probably not totally responsible for the improvement in aortic structure observed in the present study. Finally knock-out mice for Fragile X syndrome, the most frequent form of hereditary mental retardation, that are housed under standard conditions, show behavioral and neuronal abnormalities which disappear when the mice are transferred to enriched conditions [17]. In this case the enrichment consisted of a shelf, a ladder, tubes, nesting material and a running wheel, similar to the enrichment provided in the present study.

There is one major difference between the studies described above, and the present experiment. In the other studies, the manifestations of the genetic deficiencies were all neural in nature, and so it is not surprising that the environment affected the degree of manifestation of the deficiencies, since the environment is perceived by the nervous system. In the present study, arterial pathologies in fibulin-4^+/−^ mice were reduced by improved housing conditions, implying that the environment can counteract the effect of a genetic deficiency that is not directly related to perception of the environment. This process could take place by signals from the nervous system causing systemic release of hormones that bind to transcription factors and change gene expression [18]. Alternatively, environmental factors may change the methylation pattern of genes, thus altering their transcriptional capabilities [18].

This study may also have important implications with regard to humans. For example, Marfan syndrome, a connective tissue disorder in which the walls of major arteries are weakened, leading to aneurysm, has been linked to mutation of the FBN1 gene on chromosome 15 that encodes for fibrillin-1, which is an essential component of elastic fibers in connective tissue [19], [20]. Perhaps a different environment in which mild exercise is encouraged, could also reduce the debilitating effects of low levels of fibrillin-1, and decrease the incidence of aneurysm. Epidemiological studies recording the physical activity status of patients suffering aneurysms prior to the event could answer this important question and perhaps lead to preventative strategies for people who are genetically predisposed to aneurysms.
